# Intestinal malrotation in a female newborn affected by Osteopathia Striata with Cranial Sclerosis due to a *de novo* heterozygous nonsense mutation of the* AMER1* gene

**DOI:** 10.1186/s13052-022-01403-6

**Published:** 2022-12-29

**Authors:** Gregorio Serra, Vincenzo Antona, Maria Rita Di Pace, Mario Giuffrè, Giusy Morgante, Ettore Piro, Roberto Pirrello, Sergio Salerno, Ingrid Anne Mandy Schierz, Vincenzo Verde, Giovanni Corsello

**Affiliations:** Department of Health Promotion, Mother and Child Care, Internal Medicine and Medical Specialties “G. D’Alessandro”, University of Palermo, Palermo, Italy

**Keywords:** OS-CS, Skeletal dysplasia, X-inactivation, Next generation sequencing, Case report

## Abstract

**Background:**

Osteopathia Striata with Cranial Sclerosis (OS-CS), also known as Horan-Beighton Syndrome, is a rare genetic disease; about 90 cases have been reported to date. It is associated with mutations (heterozygous for female subjects and hemizygous for males) of the *AMER1* gene, located at Xq11.2, and shows an X-linked pattern of transmission. Typical clinical manifestations include macrocephaly, characteristic facial features (frontal bossing, epicanthal folds, hypertelorism, depressed nasal bridge, orofacial cleft, prominent jaw), hearing loss and developmental delay. Males usually present a more severe phenotype than females and rarely survive. Diagnostic suspicion is based on clinical signs, radiographic findings of cranial and long bones sclerosis and metaphyseal striations, subsequent genetic testing may confirm it.

**Case presentation:**

Hereby, we report on a female newborn with frontal and parietal bossing, narrow bitemporal diameter, dysplastic***, ***low-set and posteriorly rotated ears, microretrognathia, cleft palate, and rhizomelic shortening of lower limbs. Postnatally, she manifested feeding intolerance with biliary vomiting and abdominal distension. Therefore, in the suspicion of bowel obstruction, she underwent surgery, which evidenced and corrected an intestinal malrotation. Limbs X-ray and skull computed tomography investigations did not show cranial sclerosis and/or metaphyseal striations. Array-CGH analysis revealed normal findings. Then, a target next generation sequencing (NGS) analysis, including the genes involved in skeletal dysplasias, was performed and revealed a *de novo* heterozygous nonsense mutation of the *AMER1* gene. The patient was discharged at 2 months of age and included in a multidisciplinary follow-up. Aged 9 months, she now shows developmental and growth (except for relative macrocephaly) delay. The surgical correction of cleft palate has been planned.

**Conclusions:**

Our report shows the uncommon association of intestinal malrotation in a female newborn with OS-CS. It highlights that neonatologists have to consider such a diagnosis, even in absence of cranial sclerosis and long bones striations, as these usually appear over time. Other syndromes with cranial malformations and skeletal dysplasia must be included in the differential diagnosis. The phenotypic spectrum is wide and variable in both genders. Due to variable X-inactivation, females may also show a severe and early-onset clinical picture. Multidisciplinary management and careful, early and long-term follow-up should be offered to these patients, in order to promptly identify any associated morbidities and prevent possible complications or adverse outcomes.

## Background

Osteopathia Striata with Cranial Sclerosis (OS-CS) (OMIM #300373), also known as Horan-Beighton Syndrome, is a rare genetic disease [1]. It was firstly described by Voorhoeve in 1924 [2], and then named by Fairbank in 1935 [3]. About 90 patients have been reported to date [4]. OS-CS is associated with truncating point mutations or whole gene deletions of *WTX* (Wilms tumor gene, also known as *FAM123B* or *AMER1*), located at Xq11.2 [5, 6]. It shows X-linked pattern of inheritance, and distinct phenotypes based on gender [6–9]. The age at diagnosis is highly variable as well as the severity of symptoms, ranging from asymptomatic cases occasionally disclosed in adulthood, to lethal ones observed in the neonatal period. We report on a female newborn with facial and cranial characteristic features, cleft palate, hearing loss, rhizomelic shortening of lower limbs; soon after birth, she manifested some clinical signs compatible with bowel obstruction. A surgical operation has been performed, which evidenced and corrected an intestinal malrotation. The target next generation sequencing (NGS) analysis of a panel of genes involved in neurodevelopmental disorders and skeletal dysplasias detected a *de novo* heterozygous mutation of the *AMER1* gene, for diagnosis of Osteopathia Striata with Cranial Sclerosis.

## Case presentation

**Fig. 1:**
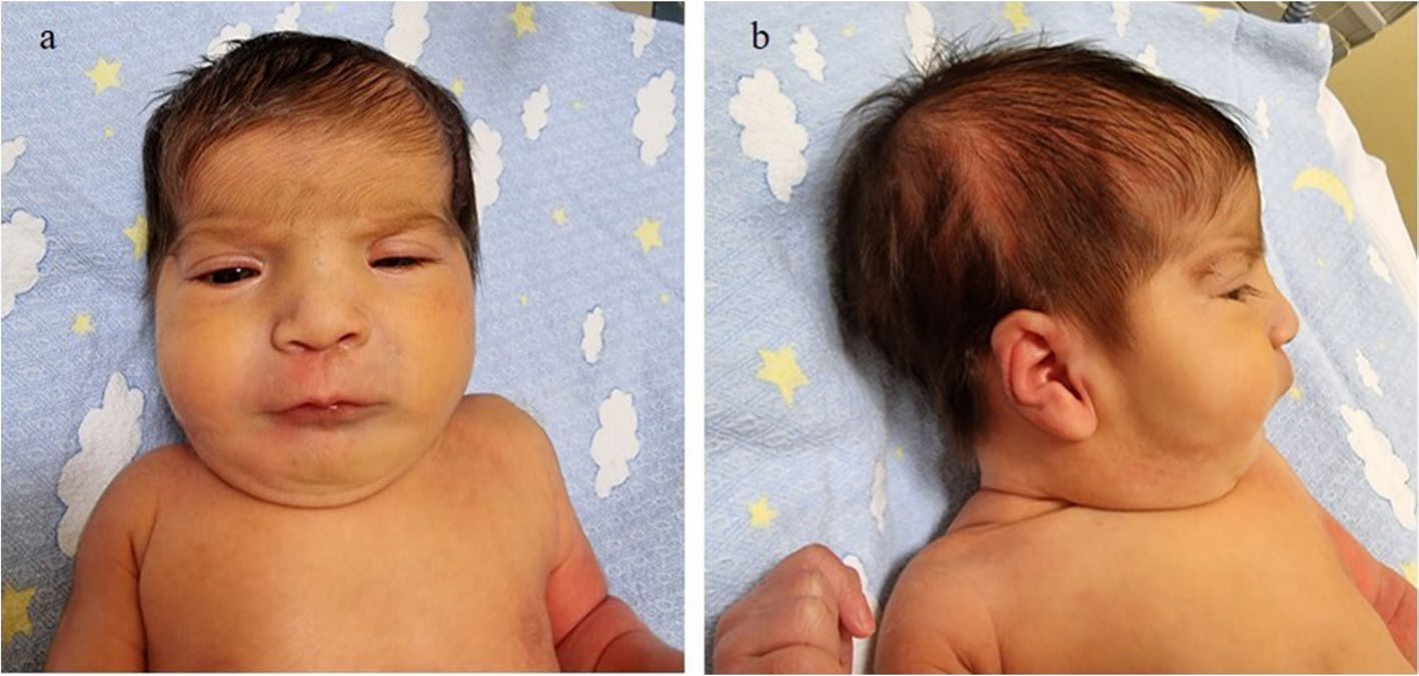
**a** Patient’s front view: frontal bossing, narrow bitemporal diameter, low anterior hairline, facial asymmetry due to left hypoplasia, hypertelorism, epicanthal folds, palpebral ptosis of the left eye, horizontal palpebral fissures, prominent nose, long philtrum and thin lips. **b** Lateral view: parietal bossing, dysplastic***, ***low-set and posteriorly rotated ear, microretrognathia

**Fig. 2:**
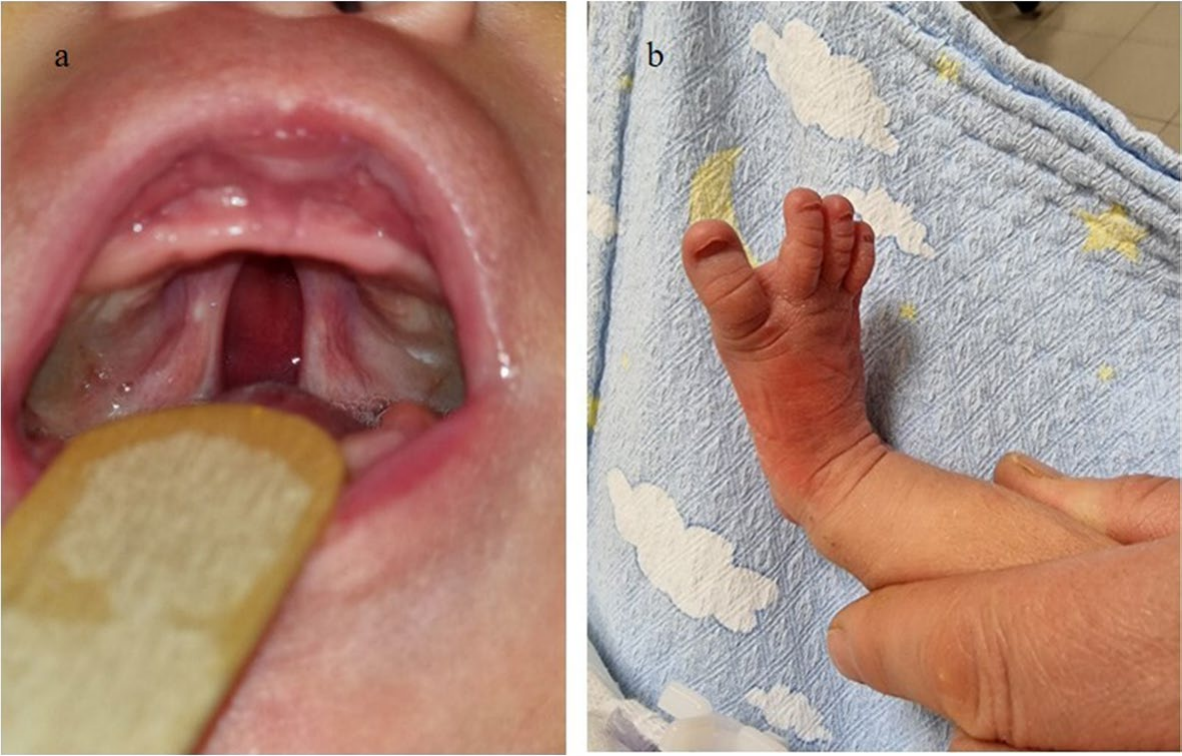
**a** Cleft of the secondary palate; **b** Sandal gap deformity between the first and second toe

**Fig. 3:**
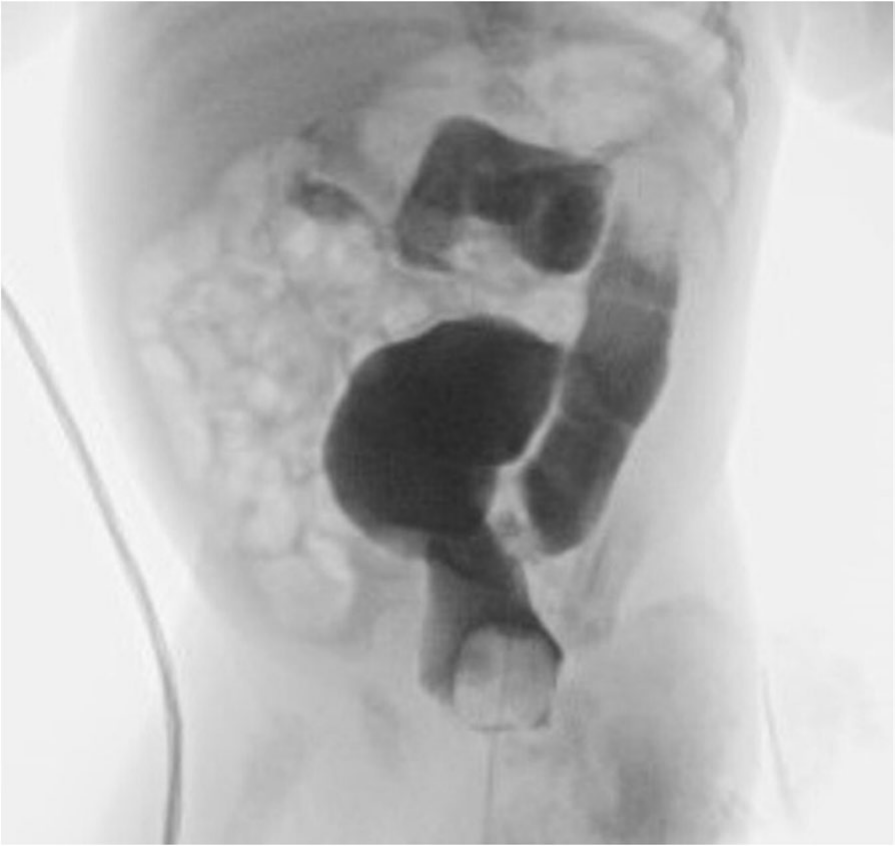
Barium enema: distension of rectum and sigma, and poor opacification of the remaining colon sections which appeared twisting and displaced to the left

A female newborn, second child of healthy and nonconsanguineous parents, was born at term by elective caesarean delivery. Due to increased age of the mother (35 year-old), a non-invasive prenatal testing through cell free fetal DNA analysis on maternal blood was performed in the first trimester of gestation, showing no chromosomal abnormalities. Further invasive genetic analyses (chorionic villus sampling and amniocentesis) were refused by parents. Prenatal ultrasound (US) investigations disclosed no anomalies until the third trimester of pregnancy (32 weeks), when severe polyhydramnios along with narrow bitemporal diameter and frontal bossing were observed. Then, a fetal magnetic resonance imaging was carried out, confirming the alterations already documented by US, and revealing no further defects. Apgar scores were 7 and 9, at 1 and 5 min respectively. At birth, anthropometric measures were as follows: weight 3300 g (61^st^ centile, + 0.28 standard deviations, SD), length 45 cm (2^nd^ centile, -2.08 SD) and occipitofrontal circumference (OFC) 33 cm (23^rd^ centile, -0.74 SD). Postnatally, due to respiratory distress, she underwent non-invasive ventilation support through nasal continuous positive airway pressure (CPAP), suspended about 24 h later. On the fifth day of life, owing to feeding difficulties and biliary vomiting, along with characteristic facial features and cleft palate, the newborn was transferred to our Neonatal Intensive Care Unit from a first level birthing center. At admission, physical examination showed frontal and parietal bossing, narrow bitemporal diameter, low anterior hairline, facial asymmetry due to left hypoplasia, hypertelorism, epicanthal folds, palpebral ptosis of the left eye, horizontal palpebral fissures, prominent nose, long philtrum and thin lips (Fig. 1a). Dysplastic, low-set and posteriorly rotated ears and microretrognathia (Fig. 1b) completed her craniofacial profile. Cleft of the secondary palate, rhizomelic shortening of lower limbs and bilateral sandal gap deformity between the first and second toe (Fig. 2a/b) were also observed. Neurological examination documented normal findings. The clinical course was characterized by inspiratory tirage during crying with short hypoxemia episodes, for which a few days oxygen supplementation was needed. X-Ray investigation and then barium enema (with 50% dilution of sodium and meglumine amidotrizoate used as contrast medium) were also performed, due to feeding difficulties, persistent biliary gastric secretions and abdominal distension. Distension of rectum and sigma, poor opacification of the remaining colon sections, which appeared twisted and displaced to the left, together with gas distension of the remaining intestinal loops shifted to the right (Fig. 3) were detected. Therefore, a laparotomy was performed, which revealed dilated and left positioned transverse and ascending colon, and the cecum located in mesogastrium, according with intestinal malrotation. A possible duodenal stenosis was ruled out. Postoperative evolution was regular, with progressive increase and tolerance of enteral nutrition. Laboratory analyses including complete blood count, serum electrolytes, liver and kidney function tests showed normal results, as well as head, heart and abdominal ultrasound (US), with the exception of duplicated gallbladder. Ophthalmological evaluation documented no abnormalities. Conversely, hearing screening through transient evoked otoacoustic emissions (TEOAE) revealed abnormal results. In order to ascertain and characterize the hearing loss, an audiological assessment was started. It included serial auditory brainstem response (ABR) evaluations at 2 and 4 months of age, which detected right response threshold at 70 dB (decibel) HL (hearing level) and left one at 60 dB, according with moderate hypoacusis, which has not required any treatment to date. Limbs X-ray, carried out during the first month of life, identified femur hypoplasia (81 mm in length, the same as for tibia, with 1:1 ratio), according with the pediatric radiology criteria for lower-limb abnormalities (i.e., the normal difference between the femur and tibia length should be 12 mm at birth, with the tibia remaining at a constant length of 80% of femoral length throughout growth [10]), and no other abnormalities. Cranial computed tomography scan, later performed, showed no alterations, ruling out craniosynostosis (cephalic index 81, obtained as transverse diameter [TD]/anteroposterior diameter [APD] × 100, normal values 75–90; APD 137 mm, TD 111 mm). Array comparative genomic hybridization (a-CGH) was performed and detected no genomic rearrangements. Then, the NGS analysis of a panel of genes involved in skeletal malformations and neurodevelopmental disorders was carried out. A heterozygous nonsense mutation of the *AMER1* gene (c.1072C > T) (Ref Seq NM_152424.3, based on genome build GRCh37/hg19; rsID 137,852,217; ClinVar:RCV000011454.5) was found, causing a premature stop codon at position 358 (p.Arg358Ter) of the encoded protein, out of a total of 1136 amino acids (condition A among the pathogenicity criteria of the American College of Medical Genetics, ACMG). This variant has been reported in literature as pathogenic in different affected female subjects and in a male fetus [5, 6]. It is extremely rare, and allelic frequency information in the dedicated population database (ExAC, EVS and 1000 Genome Project) is not available (condition G of ACMG); moreover, it is localized in a moderately conserved nucleotide position (phyloP-Vertebrate = 0.17/6.42; phyloP-Primate = 0.53/0.65; PhastCons = 0.95/1.00) (condition K). Gene sequencing was then extended to parents, who showed normal results, confirming the *de novo* origin of the genomic abnormality (condition C). Based on the clinical and genetic findings (the latter strengthened by the presence of the aforementioned pathogenicity criteria, necessary for the definition of a variant as pathogenic [11]), an OS-CS diagnosis was made. The infant was discharged at 2 months of age in good general conditions, despite poor weight and length growth, and included in a multidisciplinary (audiological/otolaryngological, ophthalmological, surgical, neurodevelopmental, orthopedic) follow-up. She is currently 9 months and 10 days old, she presents severe growth failure—according to World Health Organization growth chart for neonatal and infant close monitoring [12]—and relative macrocephaly compared to the other anthropometric measures: weight 6490 g (2^nd^ centile, -2.06 SD), length 65 cm (1^st^ centile, -2.29 SD) and OFC 44.5 (66^th^ centile, + 0.40 SD). The patient has normal muscular tone and reflexes, and developmental delay due to language and cognitive impairment. She can stand upright with support, turn her head, follow and reach objects (red cube and suspended red ring put on the midline) with both her hands. However, no lallation or attribution of meaning to gestures are currently present. She is enrolled in a specific habilitation treatment including logopedic therapy, and the surgical correction of cleft palate is already planned. She presently shows no further abnormalities.

## Discussion and conclusions

OSCS syndrome is a rare X-linked disorder. Male subjects show a wide clinical spectrum, ranging from mild to severe phenotypes. Mildly affected males show clinical features similar to affected females: male subjects may also suffer from scoliosis (which can be progressive) and joint contractures [13]. Severe phenotypes include lethal cases, due to multiple malformations such as skeletal defects, congenital heart disease, brain, genitourinary, and gastrointestinal anomalies [14–16]. Among the latter, omphalocele is the most commonly observed, followed by intestinal malrotation, which is then poorly documented in female patients like ours [4]. A striking finding in most of severely affected males is the bilateral absence or hypoplasia of the fibulae. Conversely, our female newborn shows femur hypoplasia.

Most females present macrocephaly and characteristic facial features (frontal bossing, hypertelorism, epicanthal folds, depressed nasal bridge). Indeed, our patient manifests most of the peculiar signs of the syndrome, although she actually presents a relative macrocephaly compared to the weight and length failure.

Hearing loss, cleft palate and mild developmental delay may be also observed, while radiographic findings of metaphyseal striations of long bones and cranial sclerosis are pathognomonic [4]. Both conductive and sensorineural hearing loss have been reported; hypoacusis occurs in approximately 50% of females [5, 6], and such defect has been documented also in our proband. Roughly 25% of affected females has a structural heart defect. Patent ductus arteriosus, atrial and ventricular septal defects are the most frequently described cardiac anomalies [5]. However, in our newborn case heart US revealed no abnormalities. Rarely, compression of the optic nerve due to bony sclerosis and leading to vision loss, as well as facial palsies have been reported [4].

Linear striae of long bones usually do not cause symptoms, and typically appear first between five months and six years of age [9]. In our case skeletal X-Rays and skull CT have not revealed such abnormalities to date, according to literature data. Indeed, the AMER1 protein acts on a range of cellular processes including body axis patterning and bone morphogenesis. Its reduced expression (e.g., through a loss-of-function variant) causes upregulation of osteoblastic function, leading to bony sclerosis [4]. Metaphyseal striations may be due to the presence of two independently acting osteoblast-cell lines. This can occur either through differential X-chromosome inactivation in a female, or mosaicism for an *AMER1* pathogenic variant in a male subject. This hypothesis might explain why constitutionally affected males, with a hemizygous *AMER1* pathogenic variant and only one osteoblast cell line, do not have metaphyseal striations on radiographs [4].

To achieve the diagnosis, family history as well as maternal one, showing previous miscarriages particularly of male fetuses and/or polyhydramnios (the latter present also in our case), may be useful. In addition, careful evaluation of characteristic facial features is necessary to promptly raise the diagnostic suspicion. Indeed, no consensus clinical diagnostic criteria for OS-CS have been published, but the combination of macrocephaly, cranial sclerosis, and longitudinal metaphyseal striations of the long bones are considered highly characteristic of this condition [4].

The differential diagnosis includes syndromes with cranial malformations and skeletal dysplasia, including osteopetrosis and related disorders, sclerosing bone disorders, multiple-malformation syndromes (for males with a severe phenotype), and Voorhoeve disease (characterized by isolated metaphyseal striations, and distinct from OS-CS due to the absence of cranial sclerosis or macrocephaly) [4].

Target NGS analysis is necessary to confirm the diagnosis and to identify the specific genomic pathogenic variant as well. To date, almost all reported pathogenic variants causing OS-CS are germline truncating variants or whole-gene deletions [4]: indeed, also the present genomic anomaly is a nonsense mutation. The recognition of the precise genomic profile is necessary for an accurate genetic counselling to family members (recurrence risk in case of mutations transmitted by affected mothers), in view of a possible preimplantation and/or prenatal diagnosis (primary prevention) of the disease, and in order to suggest genotype–phenotype correlations. In presence of severe mutations, as nonsense variants like in our proband, some serious clinical involvement and/or additional malformations (even the less commonly reported as the intestinal malrotation here described) may be expected. Early identification of affected subjects is crucial, especially for those who would benefit from early diagnosis and treatment of hearing and vision losses and congenital heart disease.

Some associations of OS-CS with cancer have been reported as well. To date, females with Wilms tumor [4, 17], hepatoblastoma [4, 18], and adult-onset colorectal [4, 6] and ovarian cancers [4, 6] have been observed. The association of OS-CS with chromosomal abnormalities including X chromosome has been also documented, more precisely the co-occurrence with Klinefelter syndrome [19]. Thus, these patients need careful oncologic surveillance, in addition to evaluation of congenital anomalies, especially for males [4]. However, the latter may occur also in females, due to lionization and X-inactivation mechanisms leading to variable and also severe phenotypes, even in neonatal age, like the present case. The clinical care team of patients should also include craniofacial surgeons for cleft palate, along with audiologists and ophthalmologists for periodic evaluations, owing to the increased risk of hearing loss and ophthalmoplegia, respectively.

The current database should be updated with the genomic and phenotypic findings of the present patient, in order to provide a better characterization of such a rare disease. Additional patients and the identification of new mutations will increase the knowledge on the molecular bases and the pathogenic mechanisms underlying OS-CS [20–29]. Neonatologists and pediatricians should rise such diagnostic suspicion in case of newborns/infants with macrocephaly (even if relatively severe when compared to weight and length, as in our *proposita*), peculiar facial features and limbs anomalies. Clinicians must be aware of the possibility that also female patients may have severe phenotypes and/or additional malformations, including gastrointestinal defects like omphalocele or malrotation, as in our case study [30]. Therefore, we strongly recommend a careful, early and long-term follow-up of these patients, also according with an increased risk of tumor, developmental delay, sensorial and musculoskeletal defects. Early detection of any possible associated morbidities may decrease mortality rate and adverse outcomes, it may also improve the quality of life of the affected children and their families [31–40].

## Data Availability

The datasets used and analyzed during the current study are available from the corresponding author on reasonable request.

## Abbreviations

a-CGH: Array comparative genomic hybridization
ABR: Auditory brainstem response
ACMG: American College of Medical Genetics
*AMER1*: APC Membrane Recruitment Protein 1
CPAP: Continuous Positive Airway Pressure
CT: Computed tomography
NGS: Next Generation Sequencing
OS-CS: Osteopathia Striata with Cranial Sclerosis
US: Ultrasound
TEOAE: Transient evoked otoacoustic emissions
*WTX*: Wilms tumour gene on the X-chromosome
